# Direct patterning of gold nanoparticles using flexographic printing for biosensing applications

**DOI:** 10.1186/s11671-015-0835-1

**Published:** 2015-03-12

**Authors:** Jamie Benson, Chung Man Fung, Jonathan Stephen Lloyd, Davide Deganello, Nathan Andrew Smith, Kar Seng Teng

**Affiliations:** Multidisciplinary Nanotechnology Centre, College of Engineering, Swansea University, Singleton Park, Swansea, SA2 8PP UK; Welsh Centre for Printing and Coating, College of Engineering, Swansea University, Singleton Park, Swansea, SA2 8PP UK; College of Science, Department of Physics, Swansea University, Singleton Park, Swansea, SA2 8PP UK

**Keywords:** Surface patterning 81.65.Cf, Nanostructured materials in electrochemistry 82.45.Yz, Biosensors 87.85.fk, AuNPs, Gold, Nanoparticle, Flexographic, Printing, Ink, Biosensing, Glucose

## Abstract

In this paper, we have presented the use of flexographic printing techniques in the selective patterning of gold nanoparticles (AuNPs) onto a substrate. Highly uniform coverage of AuNPs was selectively patterned on the substrate surface, which was subsequently used in the development of a glucose sensor. These AuNPs provide a biocompatible site for the attachment of enzymes and offer high sensitivity in the detection of glucose due to their large surface to volume ratio. The average size of the printed AuNPs is less than 60 nm. Glucose sensing tests were performed using printed carbon-AuNP electrodes functionalized with glucose oxidase (GOx). The results showed a high sensitivity of 5.52 μA mM^−1^ cm^−2^ with a detection limit of 26 μM. We have demonstrated the fabrication of AuNP-based biosensors using flexographic printing, which is ideal for low-cost, high-volume production of the devices.

**Electronic supplementary material:**

The online version of this article (doi:10.1186/s11671-015-0835-1) contains supplementary material, which is available to authorized users.

## Background

Noble metal nanoparticles (NPs) have unique electrical, optical, thermal and catalytic properties, which find applications in non-toxic drug delivery systems [1], molecular biosensing [2], cellular imaging [3] and protein detection [4] among others. They have many advantages over other nanomaterials, as they are more stable and conductive [5]. These noble metal NPs can range in sizes from 1 to 100 nm and can be differently shaped [6]. They also have the added advantage of being biocompatible [7]. These types of NPs have been commonly used in molecular diagnostics as they offer excellent sensitivity due to their very large surface to volume ratio and ease of functionalisation for the detection of specific analytes in biological solutions [7]. Among the most extensively used are gold nanoparticles (AuNPs). They have been used in many nanotechnology-based biosensors due to the biocompatibility of the material in addition to its many unique optical and electrical properties. An example of AuNP-based biosensors can be seen in work carried out by Feng et al. where an electrochemical deposition method was used to deposit an AuNP-chitosan film which was subsequently used for the determination of glucose [8]. Another example is work performed by Jena et al. where they introduced AuNPs to a 3D silicate network using a sol-gel process [9]. This was utilised as an enzyme-free biosensor for glucose detection. Their sensor showed high sensitivities of 0.179 nA nM^−1^ cm^−2^ and showed great stability and reproducibility. AuNPs have also been used extensively in enzymatic biosensors. An example of this can be seen in the work of Zhang et al., where they fabricated a glucose sensor using dithiol immersion of a gold electrode followed by immersion in cystamine and AuNPs with subsequent attachment of glucose oxidase (GOx) [10,11]. Sensors developed in this way showed good sensitivities of 8.8 μA mM^−1^ cm^−2^ [10] and 8.3 μA mM^−1^ cm^−2^ [11]. These sensors highlight the use of AuNPs in highly sensitive biosensors for the detection of not just glucose but a multitude of other biomarkers. However, current methods in fabricating AuNP electrodes are very laborious and time-consuming and are therefore not suitable for scaled-up production of biosensors. This highlights a significant market gap for methods that lend themselves to mass production of AuNP electrodes and thus rendering the device commercially viable. Flexographic printing techniques could overcome these restrictions and help bring highly sensitive AuNP-based sensors to the mass market at low cost in comparison to other techniques. The ability for patients to test themselves for a range of conditions within the comfort of their own home is highly attractive. The point-of-care (PoC) diagnostic device market is poised to reach $27.5 billion by 2018, with a wide range of PoC technologies covering many different diseases and conditions [12]. In order to ensure commercial viability of these technologies, there is a requirement for low cost, high yield fabrication of such devices. The use of printing techniques is an obvious step towards mass production of devices at a relatively low cost when compared to the use of semiconductor cleanroom techniques, which involve multiple processing steps using complex and expensive facilities. Various printing techniques, such as screen printing [13] and inkjet printing [14], have been utilised for biosensor fabrication. Herein, we report the novel use of flexographic printing techniques in the fabrication of AuNP-based devices, such as an electrochemical biosensor. The ability to incorporate many printing rolls allows the printing of multiple layers consecutively via a straightforward printing process. Furthermore, it does not suffer from the ‘coffee-ring’ effect or clogging of printing heads as observed in the inkjet printing technique [15]. The flexographic printing technique offers a simple and rapid fabrication process for AuNP-based devices.

In this work, carbon electrodes and AuNPs were printed onto a polyimide substrate through the use of flexographic printing. Here, an AuNP ink was developed for the printing technique. Glucose sensors were fabricated, as an exemplar biosensor, to demonstrate the viability of using the flexographic printing technique in the production of AuNP-based electrochemical biosensors. Glucose sensing was chosen due to its popularity and large-scale clinical relevance. The use of GOx for the enzymatic detection of glucose has been utilised for electrochemical glucose sensing for many years, on various electrode constructions, including those incorporating AuNPs. This is due to its well-established selectivity, reliability and relatively low cost [16-19]. This printed device could also be used for a multitude of other enzymatic biosensing applications through functionalisation with other enzymes as well as the wealth of other uses for AuNPs discussed above. Sensors fabricated in this work have shown high sensitivity and also displayed a low limit of detection (LoD). GOx immobilised onto the printed AuNPs at the carbon electrodes has shown very good electron transport properties indicated by the fast response time of the sensor to the presence of even small concentrations of glucose.

## Methods

### Materials

Polyimide was obtained from Katco (Katco, Milton Keynes, UK) and was cleaned via ultrasonication with acetone prior to use. GOx (259 U mg^−1^) was purchased from BBI Solutions (BBI Solutions, Cardiff, UK) and was used as received. Glucose was obtained from Fisher Scientific (Fisher Scientific, Loughborough, UK) and made into various concentrations in deionised (DI) water at least 24 h prior to sensor testing to allow for mutarotation. HAuCl_4_ · xH_2_O, polyvinylpyrrolidone (PVP), NaBH_4_, cysteamine and glutaraldehyde were purchased from Sigma-Aldrich (Sigma-Aldrich, Dorset, UK). Carbon flexographic ink was purchased from Gwent Group (Gwent Group Limited, Pontypool, UK) and used as received. Paraffin wax and Phosphate buffered saline (PBS) solution with pH 7.4 were purchased from Fisher Scientific (Loughborough, UK).

### Fabrication of AuNP ink

AuNPs were synthesised through the chemical reduction of HAuCl_4_ via NaBH_4_ addition. A volume 30 ml of DI water was stirred rapidly in a conical flask using a stirrer bar. Next, 0.2 g of HAuCl_4_ was added to the conical flask followed immediately by 0.15 g of PVP. The PVP acts as a capping agent and prevents the agglomeration of nanoparticles. The solution was allowed to stir for 20 min. A 1-ml aliquot of NaBH_4_ solution was prepared containing 0.05 g of NaBH_4_. A few drops of 1 M NaOH were added to the aliquot, and it was placed in the freezer until it reached <5°C. The NaBH_4_ solution was then added to the conical flask rapidly in two 500-μl aliquots. The nanoparticle reduction was indicated by a solution colour change from bright yellow to dark purple, and, as expected, hydrogen evolution was observed. This solution was allowed to stir for 4 h. After 4 h, the solution was poured into a centrifuge tube and centrifuged at 4,000 rpm for 30 min. After centrifugation, a large pellet of AuNPs was present at the bottom of the centrifuge tube. The supernatant was removed carefully to ensure the pellet was not disturbed. The pellet was then re-dispersed in 70% isopropyl alcohol (IPA) (14 ml) and 30% DI water (6 ml) via ultrasonication. The AuNP ink was then ready for flexographic printing. This is a simple method that only involves a small number of steps. To the knowledge of the authors, AuNP ink has not previously been fabricated through this specific method.

### Electrode preparation

**Figure 1:**
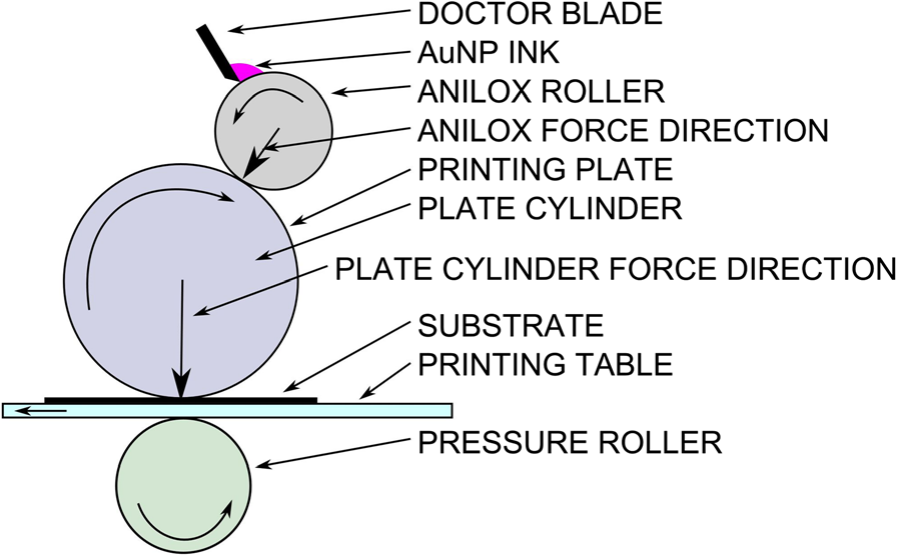
**Schematic diagram of a flexographic printer.** Ink is transferred to the printing plate from the anilox roller at a rate controlled by the anilox surface features and the doctor blade. Ink is then printed onto the substrate from the printing plate in a continuous roll-to-roll manner.

**Figure 2:**
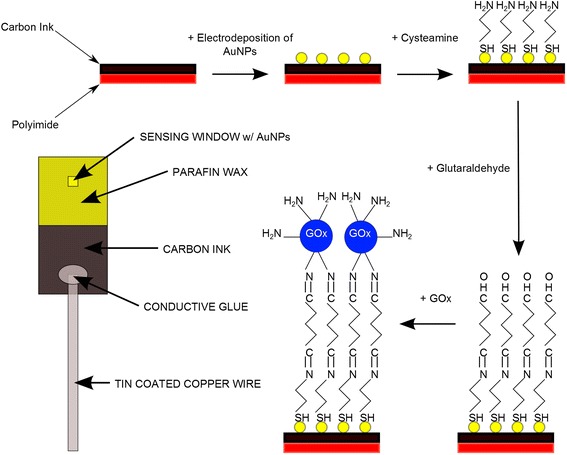
**Schematic diagram showing the functionalisation scheme.** The scheme shows the major steps in the functionalisation scheme for AuNPs on carbon electrode. Starting with the printed carbon electrode AuNPs are printed and functionalized with cysteamine, glutaraldehyde and glucose oxidase, also included is a schematic of a fully prepared electrode for biosensing (left).

Flexographic printing was performed using an IGT F1 flexographic test printer (IGT Testing Systems, Amsterdam, The Netherlands). A schematic of the system is shown in Figure 1. The working electrode was prepared by first printing carbon ink onto a sheet of polyimide using the flexographic printing technique. After printing carbon onto the polyimide substrate, the samples were annealed at 150°C in an oven for 10 min. The polyimide, with printed carbon, was then cut into 1 cm × 2 cm sections for use as an electrode. The printed carbon sections were then placed back onto the flexographic printer for AuNP printing using the ink described previously. The printing parameters for flexographic printing of our ink were optimised as follows; printing force of 125 N, anilox force of 125 N, and speed of 0.6 m s^−1^. Samples were then annealed at 150°C for 10 min to dry and remove the residual PVP from the AuNPs. Patterning of the AuNPs onto the polyimide is possible, using the flexographic printing technique, through the use of patterned printing plates. A short length of tin-coated copper wire was then attached to the carbon electrode in an area where no AuNPs had been printed to allow an electrical connection between a sample and a potentiostat. The wire was attached using a conductive epoxy. Paraffin wax was then used to define a small window (~2 mm^2^) in the AuNP-coated carbon region for functionalisation. Cysteamine and glutaraldehyde were treated onto the AuNPs prior to the functionalisation of GOx. The sensing window was treated with 5 μl of 20 mM cysteamine for 30 min after which it was washed with DI water and dried using N_2_ gas. The sensing window was then exposed to 5 μl of 4% glutaraldehyde solution for 30 min and once again washed and dried as previous. The final step in the electrode preparation was to introduce GOx to the sensing window, where 5 μl of 7 mg ml^−1^ GOx was added to the window and left overnight to ensure its attachment to the electrode surface. The window was washed thoroughly with PBS and dried under N_2._ The printed carbon-AuNP-GOx electrode was then ready for electrochemical experiments. A schematic diagram of the working electrode fabrication process is shown in Figure 2.

### Characterisation

The morphology of the AuNPs coated onto the carbon substrate was studied using a Hitachi S-4800 scanning electron microscope (SEM) (Hitachi High-Technologies Corporation, Tokyo, Japan) at 20 kV. The SEM was equipped with an Oxford Instruments energy dispersive X-ray (EDX) detector (Oxford Instruments, Oxfordshire, UK) which was subsequently used to determine the chemical composition of the printed AuNP samples. Scanning auger microscopy (SAM) and auger electron spectroscopy (AES) were carried out using an Omicron NanoSAM detector (Omicron NanoTechnology GmbH, Taunusstein, Germany) with an accelerating voltage of 5 kV, 1 nA beam current and a 90-μm aperture. Electrochemical measurements were carried out using a CompactStat (Ivium Technologies, Eindhoven, The Netherlands). All electrochemical experiments were conducted using a three-electrode system which comprised of a printed carbon-AuNP-GOx working electrode, a gold-wire counter electrode and a Ag/AgCl reference electrode. The three-electrode system was always orientated in the same manner to avoid any variation from sample to sample. All electrochemical experiments were performed at room temperature inside a Faraday cage, PBS was used as the supporting electrolyte, and experiments were conducted under constant stirring. The magnetic stirring provided the electrolyte solution with sufficient conductive transport. Chronoamperometry was performed at +0.8 V with an interval time of 1 s in an air-saturated environment and used for glucose detection. The current was allowed to settle to a constant value prior to any glucose additions.

## Results and discussion

AuNP ink formulations were tested on printed carbon electrodes fabricated as described previously. Many different ink formulations and printing parameters were explored to find the best approach. The evaporation rate and polymer content of the ink is of paramount importance. In early attempts, high IPA percentages of 80% to 95% were adopted. It was seen that the solvent would evaporate too quickly resulting in a large loss of gold on the printing plate. Addition of extra polymer to the ink was also tested aiming to improve dispersion stability. This resulted in an increased viscosity, but also decreased wetting of the carbon surface with the AuNP ink. This resulted in visibly non-uniform deposition of polymer at the surface. With 70% IPA and 30% water, the printing of the AuNP ink was relatively uniform and the AuNP distribution on the carbon surface was suitable for sensor testing. The printing force also had an effect on the quality of the printing. It was observed that high printing forces would cause the ink to spread too much, and low printing forces would result in blobs of ink at the electrode surface. The printing force was optimised to 125 N as this gave the most consistent and evenly distributed matrix of AuNPs on the surface of the carbon electrode. It was found that rapid addition of ice-cold NaBH_4_ produced smaller nanoparticles than synthesis done with room temperature NaBH_4_ added more slowly. With the synthesis parameters described above, an ink with the desired characteristics, such as small AuNPs and good viscosity, to hold the nanoparticles in suspension was achieved.

**Figure 3:**
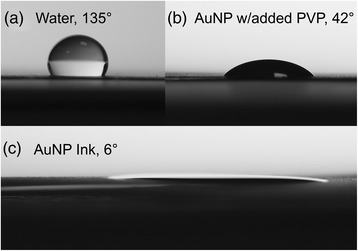
**Images showing the contact angles of inks and for comparison**, **water.** Images showing contact angles for **(a)** water, **(b)** AuNP ink + 0.5 g PVP and **(c)** AuNP ink, after drop casting onto printed carbon substrates. The images show contact angles of **(a)** 135°, **(b)** 42°, and **(c)** 6°, respectively, showing improved wetting by the AuNP ink.

Figure 3a shows the contact angle of a water droplet, which is shown as a control, after drop casting onto a printed carbon electrode. The droplet shows a contact angle of 135° indicating poor wetting of the electrode surface. In order to investigate the wetting issues seen by ink formulations with high PVP concentration, an additional 0.5 g of PVP was added to our ink and drop cast onto the printed carbon surface. Figure 3b shows the contact angle at this concentration to be 42° and indicates better wetting than water. However, the surface of the printed carbon was not completely wetted by the ink with this formulation. This resulted in inconsistent coverage where, after drying, some areas contained high densities of AuNPs and others contained low densities. This can occur throughout the printing process and can have a detrimental effect on the quality of the printed electrodes. Figure 3c shows a contact angle image of the optimised AuNP ink developed in this work which was used in the fabrication of the working electrode. The image shows the ink wets the surface well and has a very small contact angle of 6°. This demonstrates that the ink has the required wetting properties to provide consistency and uniformity in the deposition of AuNPs on the substrate.

**Figure 4:**
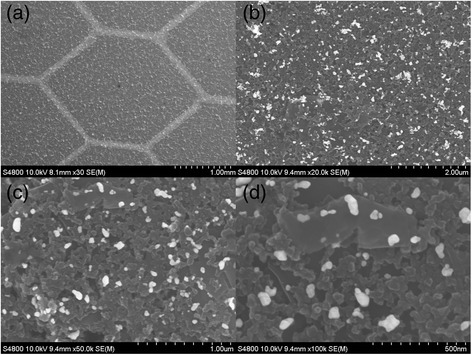
**SEM image of printed AuNP ink on carbon electrodes showing the pattering of the particles. (a)** SEM characterisation shows well-defined patterning of the AuNP ink in a printed honeycomb pattern on carbon electrodes, at low magnification (30×). Higher magnification images **(b)** 20, **(c)** 50 and **(d)** 100 kx show the even distribution of the AuNPs on the surface.

Figure 4 shows SEM images of printed AuNPs on carbon-polyimide substrate. Figure 4a demonstrates the selective patterning of AuNPs using the flexographic printing technique, producing line widths of <120 μm from a printing plate with a 100-μm width; the lines also displayed low edge distortion, and line width shows a linear relationship with printing force. The printed honeycomb pattern has well-defined edges, and this intricate pattern highlights the capabilities of the technique to perform selective patterning of the AuNPs during device fabrication. This is a highly desirable characteristic as it allows for areas of a substrate to be separated from each other. This is particularly applicable to full sensor fabrication as it allows the AuNP electrode to be electrically isolated from other electrodes on the same substrate. Figure 4b shows a uniform distribution of AuNPs on the carbon surface. There are some areas of agglomeration, but this is not deemed significant enough to have a detrimental effect on the biosensing capabilities of the electrode. Figure 4c is a 50-kx magnification image of AuNPs printed onto the substrate. The image shows limited agglomeration of the AuNPs, and the distribution of the particles is relatively uniform, which is typical of the entire printed area. Figure 4d is a 100-kx magnification image of the AuNPs, it can be seen that the size of the majority of nanoparticles is less than 60 nm.

**Figure 5:**
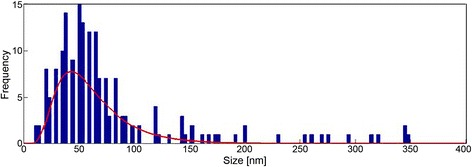
**Histogram showing AuNP size distribution on carbon electrode.** After flexographic printing of the AuNP ink, particle size analysis was carried out on a 50-kx magnification SEM image of the particles. This analysis shows that the majority of the particles are less than 60 nm in size.

Particle size analysis was carried out using MATLAB particle size distribution software on a 50-kx magnification SEM image of printed AuNPs. Figure 5 is a histogram showing size distribution of printed AuNPs on a carbon-polyimide substrate after flexographic printing using the parameters previously described. The vast majority of particles are under 60 nm, which is ideal for high-sensitivity biosensing applications due to the very large surface to volume ratio of these nanoparticles, and they favour the immobilisation of enzymes [20]. There are a very small number of particles over 100 nm, and these large particles are likely to be agglomerations of smaller particles. Due to the very small number of large particles, we can assume that they will not have a significant effect on the performance of the sensor.

A simple thermogravimetric process was carried out to assess the presence of PVP after post-annealing of the printed surface. AuNP ink was drop cast onto a substrate and dried at room temperature overnight. The sample was then weighed, annealed at 150°C for 10 min and re-weighed yielding a 62% drop in weight which corresponds to the loss of polymer from the surface. This correlates with results seen by Cui et al. where they saw a total mass loss of 57% attributed to the loss of PVP [21]. This post-annealing step to remove PVP is important for the subsequent functionalization of the electrode for biosensing applications.

**Figure 6:**
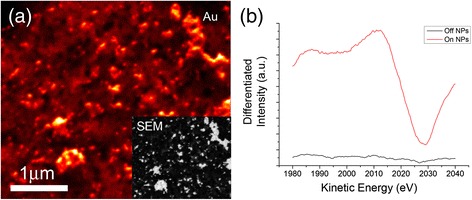
**Scanning auger microscopy analysis of printed AuNPs on carbon electrode. (a)** Scanning auger microscopy image of AuNPs using peak intensity from the centre of the Au peak (inset shows SEM image of corresponding area). **(b)** Differentiated spectra taken from areas on the AuNPs (red) and off of the AuNPs (black).

**Figure 7:**
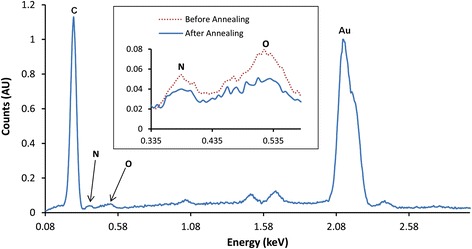
**EDX spectrum of printed AuNPs at carbon electrode before and after annealing.** Energy dispersive X-ray analysis of the AuNPs printed onto a carbon electrode shows carbon, gold, nitrogen and oxygen. The inset shows nitrogen and oxygen peaks before (red) and after (blue) annealing, indicating a reduction in nitrogen and oxygen peaks due to the annealing process.

A combination of SAM and AES analyses was used to investigate the printed AuNPs at the carbon electrode. Figure 6a shows a SAM image of the printed AuNPs on carbon electrode. The scan was performed by fixing the detector to the energy of the Au3 MNN transition and raster scanning across the scan window. The high-intensity regions correspond to gold on the substrate surface, with the darker areas representative of the printed carbon (no AuNPs). Point AES measurements were performed on and off the AuNPs as shown in Figure 6b. The red line corresponds to point spectra performed on the AuNP whereas the black line shows point spectra on bare carbon. AES performed at the bare carbon showed only one observable peak in the survey spectra (see Figure S1 of Additional file 1) at 262.5 eV, corresponding to the C1 KLL auger transition. Spectra on the AuNPs, however, show a distinctive peak at 2,016 eV, characteristic of the Au3 MNN transition. The presented AES spectra in Figure 6b have been differentiated using a 2-eV Savitzky-Golay smoothing width to enhance the signal to noise ratio without losing peak information. The AES measurements suggest that the samples comprise of only carbon and AuNPs on the surface as there are no observable peaks other than those from carbon and gold transitions. This is further supported by the energy dispersive X-ray (EDX) spectrum as shown in Figure 7. There are two large peaks which are attributed to carbon and gold at the printed AuNP-carbon electrode. The large gold peak indicates the presence of substantial gold on the carbon surface. The inset shown in Figure 7 highlights the nitrogen and oxygen peaks before and after annealing, there is a significant drop in the number of counts and this is due to the loss of PVP from the electrode surface which contains nitrogen and oxygen groups in its structure. It is important to remove PVP as it could have a detrimental effect on the performance of the device. The result shows that the printing techniques developed and employed in this work are capable of producing surfaces with AuNPs that are uniformly distributed onto the substrate, which is ideal for the fabrication of devices such as biosensors.

**Figure 8:**
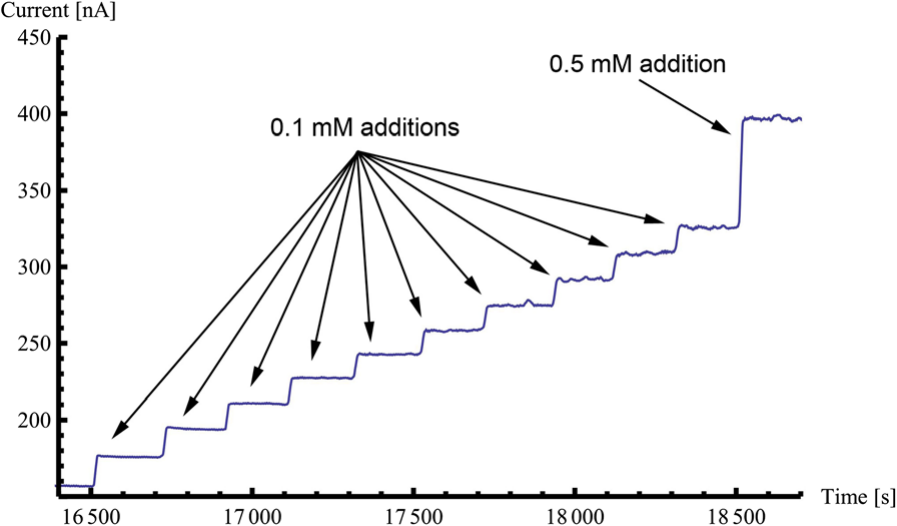
**Chronoamperometric graph showing fabricated polyimide**-**carbon**-**AuNP**-**GOx electrode response to glucose additions.** After functionalisation, samples were tested for the electrochemical detection of glucose. These samples where tested with glucose concentration step increments of 0.1 and 0.5 mM as detailed in the figure.

AuNPs provide a biocompatible environment for the use of enzymes, such as GOx. In studies using such nanoparticles, it has been shown that the activity of GOx can be enhanced [22]. Figure 8 shows a chronoamperometric graph for a typical printed polyimide-carbon-AuNPs-GOx electrode fabricated as described previously. It can be seen from the graph that there is a clear, rapid response to glucose additions. The sensor displays a 2.1-nA step for a 0.01-mM addition (see Figure S2 of Additional file 1). The sensor exhibited 18.4-nA steps for ten subsequent 0.1-mM glucose additions and a large 72.2-nA step for a 0.5-mM glucose addition. The clear steps and fast response exhibited are highly desirable in PoC diagnostic devices.

**Figure 9:**
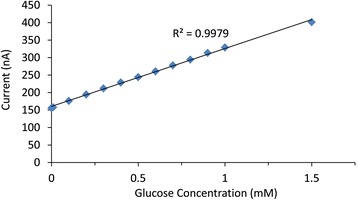
**Calibration curve for the polyimide**-**carbon**-**AuNP**-**GOx electrode responding to glucose.** Plot of steady state current against concentration of glucose taken from the chronoamperometric data for a polyimide-carbon-AuNP-GOx electrode. The graph shows the linear response of the electrode for glucose additions up to 1.5 mM.

Figure 9 is the calibration curve for the glucose sensor. The graph shows a very good linear response to glucose additions, resulting in an *R*^2^ value of 0.9979. The linear range of the sensor was from 0.01 to 1.5 mM. After this point, the device began to saturate which was indicated by a plateau beginning to form with additions after 1.5 mM. The sensor displayed a high sensitivity of 5.52 μA mM^−1^ cm^−2^ with a detection limit of 26 μM. The limit of detection was calculated using the following formula:

$$ \mathrm{L}\mathrm{o}\mathrm{D} = 3RS{D}_{\mathrm{B}}/{I}_{\mathrm{mM}} $$

(*RSD*_B_ is the relative standard deviation of blank signal, and *I*_mM_ is the current per millimole).

The sensitivity of this device is similar to those previously reported for AuNP glucose sensors functionalised through similar methods [10,11]. However, the printed device shown here has demonstrated the use of a fabrication technique which holds the potential for mass production.

This work has demonstrated the use of flexographic printing for the patterning of AuNPs onto electrode surfaces for biosensing applications. The main issue with previous work carried out on AuNPs is that much of the fabrication technique is not transferable to mass production. Glucose sensing is just one of many potential applications of this technology. The use of flexographic printing can significantly reduce the production cost of the devices as it allows the fabrication of vast numbers of devices at relatively low cost. This is due to the fast printing process with great accuracy allowing direct patterning of nanomaterials onto a substrate. This can be compared to methods employed by others whereby the attachment of AuNPs to the surface of the electrode takes a 6-h immersion in an AuNP solution [10], while flexographic printing would take seconds to print potentially hundreds of samples. Flexographic printing offers very high throughput as compared with other printing techniques, such as screen and inkjet printing. The technique is able to perform roll-to-roll printing at a speed of up to 300 m min^−1^. Inkjet printing is significantly slower and also suffers from problems such as blockage of the fine printing head and ‘coffee-ring’ effect [23]. It is clear that the printing of AuNPs via flexographic printing allows a high throughput of devices, and the sensitivities achieved on our printed AuNP glucose sensors are comparable to those seen through other fabrication methods.

## Conclusions

This work has demonstrated the direct patterning of AuNPs on substrates using flexographic printing which is ideal for volume production at a relatively low cost. Results showed that the printed AuNPs are uniformly distributed on the carbon electrode using an optimised ink formulation and printing parameters. AuNP-based glucose sensors developed using the technique display a rapid response to the addition of glucose and have a sensitivity of 5.52 μA mM^−1^ cm^−2^ with a low detection limit of 26 μM. High throughput, low-cost production of AuNP-based biosensors is highly viable, and the technology can be transferred to a wide range of biosensing applications.

## Additional file

Additional file 1:
**Supporting Information.** This file shows the supplementary AES spectrum on bare carbon and measurement on low glucose concentration.

## Abbreviations

AES: auger electron spectroscopy
AuNPs: gold nanoparticles
EDX: energy dispersive X-ray
GOx: glucose oxidase
LoD: limit of detection
NPs: nanoparticles
PBS: phosphate buffer saline
PoC: point-of-care
PVP: polyvinylpyrrolidone
SAM: scanning auger microscopy
SEM: scanning electron microscopy
