# Supplementary material for: Direct patterning of gold nanoparticles using flexographic printing for biosensing applications
Source: Nanoscale Res Lett. 2015 Mar 12;10:127. doi: 10.1186/s11671-015-0835-1 (PMC4385249; doi:10.1186/s11671-015-0835-1)
Supplement: Additional file 1: — Supporting Information. This file shows the supplementary AES spectrum on bare carbon and measurement on low glucose concentration. [file 11671_2015_835_MOESM1_ESM.docx]

(Supporting Information)

Direct patterning of gold nanoparticles using flexographic printing for biosensing applications

Jamie Benson^1^, Chung Man Fung^1^, Jonathan S.Lloyd^1^, Davide Deganello^2^, Nathan A. Smith^3^ and Kar Seng (Vincent) Teng^1 *^

1 Multidisciplinary Nanotechnology Centre, College of Engineering, Swansea University, Singleton Park, Swansea SA2 8PP, United Kingdom 
2 Welsh Centre for Printing and Coating, College of Engineering, Swansea University, Singleton Park, Swansea SA2 8PP, United Kingdom

3 College of Science, Department of Physics, Swansea University, Singleton Park, Swansea SA2 8PP, United Kingdom

KEYWORDS - AuNPs, biosensing, flexographic, printing, ink, glucose, gold, nanoparticle

*To whom correspondence should be addressed - K.S.Teng@swansea.ac.uk


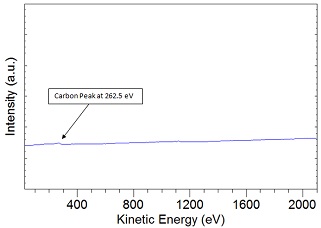


Figure S1 – AES survey spectrum on bare carbon electrode showing one observable peak at 262.5eV


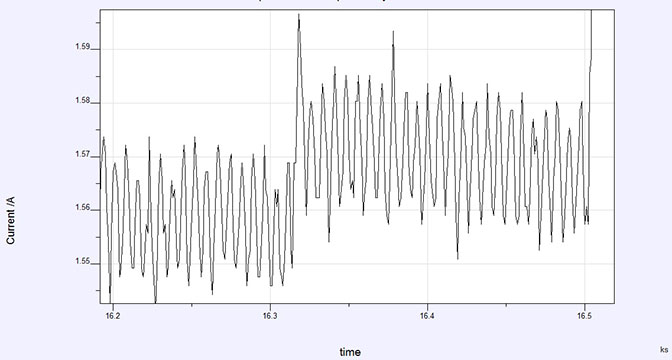


Figure S2 – Chronoamperometric graph showing ~2.1nA step for 0.01 mM glucose addition to functionalised AuNP electrode
